# Post-intravitreal injection endophthalmitis secondary to *Turicella otitidis*: a case report

**DOI:** 10.1186/s12886-020-01412-1

**Published:** 2020-04-10

**Authors:** Danny A. Mammo, Daniel Watson, Karen R. Armbrust

**Affiliations:** 1 Department of Ophthalmology and Visual Neurosciences, University of Minnesota, 420 Delaware Street Southeast, MMC 493, Minneapolis, MN 55455 USA; 2 Department of Ophthalmology, Veterans Affairs Health Care System, Minneapolis, MN USA

**Keywords:** Endophthalmitis, Anti-VEGF, Aflibercept, Turicella otitidis, Case report

## Abstract

**Background:**

Endophthalmitis is a rare but potentially devastating complication of intravitreal injection. The causative organism plays an important role in prognosis following endophthalmitis. Here we present the first reported case of *Turicella otitidis* endophthalmitis, which is notable for a delayed presentation.

**Case presentation:**

A 71 year old male who was receiving intravitreal aflibercept injections for neovascular age-related macular degeneration presented 4 weeks after his most recent intravitreal injection and was found to have endophthalmitis. Polymerase chain reaction (PCR) testing of aqueous fluid was positive for *Turicella otitidis*. The endophthalmitis responded well to treatment with intravitreal antibiotics.

**Conclusions:**

Coryneform bacteria are a rare cause of endophthalmitis, and this is the first reported case of endophthalmitis caused by the corynebacterium species *Turicella otitidis*. As in this case, post-intravitreal injection endophthalmitis may have a bacterial etiology even with delayed presentation. The relatively indolent disease course and excellent response to intravitreal antibiotics is consistent with previous ophthalmic reports regarding other corynebacteria, as well as with otolaryngology and hematology oncology reports addressing *Turicella otitidis* specifically. This case supports the growing body of evidence for pathogenicity of *Turicella otitidis* and demonstrates the utility of PCR for diagnosis in small volume aqueous specimens.

## Background

Intravitreal anti-vascular endothelial growth factor (VEGF) agents have transformed care for many vitreoretinal disorders, allowing visual improvement or stability in diseases with previously poor visual outcomes, such as age-related macular degeneration (AMD) with associated choroidal neovascularization (CNV). However, endophthalmitis remains a rare but potentially devastating complication of anti-VEGF intravitreal injection, with rates of post-injection endophthalmitis ranging from 1 case in 1000 to 1 in 6450 [1–4]. The majority of culture-positive endophthalmitis cases are coagulase-negative *Staphylococcus* species, which usually are associated with good outcomes. Poorer clinical outcomes have been reported in post-anti-VEGF endophthalmitis associated with other species, particularly in *Streptococcus* species. Corynebacterium, a genus of gram positive bacilli or coccobacilli, is a rare cause of post-procedural endophthalmitis [5–7]. Corynebacterium was found in only about 1% of the Endophthalmitis Vitrectomy Study culture positive endophthalmitis cases [8] and has only rarely been reported after anti-VEGF injection [2]. This is the first report of Corynebacterium species *Turicella otitidis* endophthalmitis following an ocular procedure. We review the patient’s presentation, diagnosis, and response to treatment.

## Case presentation

**Fig. 1:**
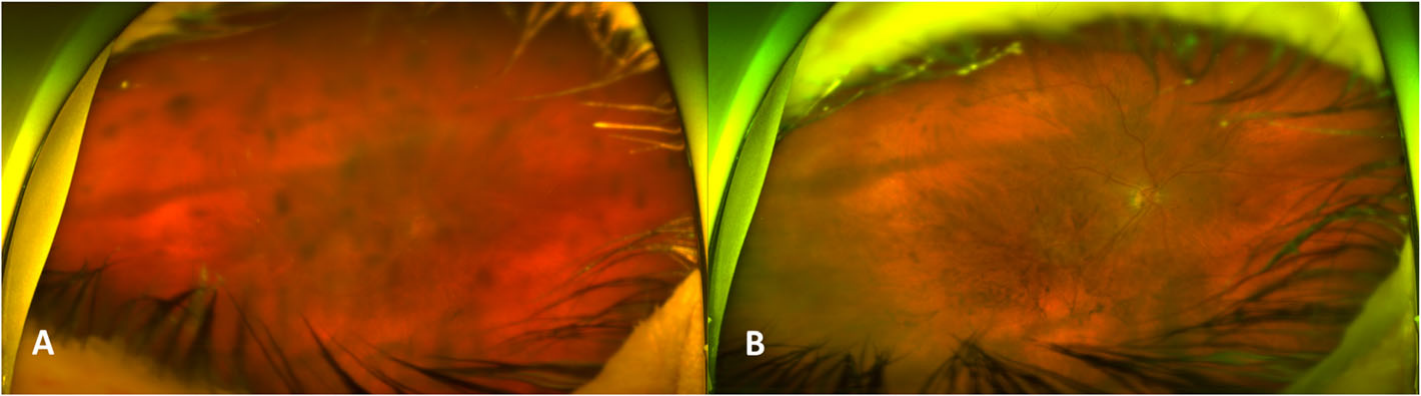
Optos wide-field fundus imaging of the right eye **a**) at presentation showing dense vitreous debris and **b**) 4 weeks after intravitreal antibiotics showing near resolution of the vitreous debris

A 71 year old male with a history of exudative AMD status post 14 intravitreal aflibercept injections, right eye, and non-exudative AMD, left eye, presented to clinic for his scheduled intravitreal aflibercept injection. His most recent anti-VEGF treatment was 4 weeks prior to presentation. At presentation, the patient reported gradually worsening cloudy vision, new floaters, and photophobia right eye as well as a dull ache behind his right eye, managed with over the counter pain medication. On examination, his visual acuity, right eye, was 20/250, decreased from his baseline of 20/60. Slit lamp examination of the right eye revealed conjunctival injection, confluent granulomatous keratic precipitates, grade 4+ anterior chamber cell, and grade 2+ flare without fibrin. There was mild nuclear sclerosis in both eyes, symmetric between the two eyes. Dilated fundus exam showed vitreous haze without obvious retinitis in the right eye (Fig. 1a) and macular drusen left eye. B-scan ultrasonography of the right eye demonstrated scattered vitreous opacities with increased opacity concentration temporally, temporal chorioretinal thickening, and no retinal or choroidal detachment. Fluorescein angiography of the right eye was limited by poor image quality, but the focal area of hyperfluorescence in the temporal macula was more consistent with the pre-existing choroidal neovascular membrane rather than an abscess.

Most patients with post-injection bacterial endophthalmitis present within 1 week of the causative injection [9, 10], so a broad differential diagnosis was entertained for this relatively indolent panuveitis. A bacterial exogenous endophthalmitis still was considered the most likely diagnosis, but blood cultures were drawn from 2 separate sites and serum testing for Quantiferon-Tb, rapid plasma reagin (RPR), fluorescent treponemal antibody absorption (FTA-Abs), Lyme enzyme immunoassay, Toxoplasma immunoglobulin M and immunoglobulin G, angiotensin converting enzyme (ACE), and antineutrophil cytoplasmic antibodies (ANCA) was performed to rule out other causes of uveitis. The patient underwent treatment with empiric intravitreal vancomycin (1 mg in 0.1 ml) and ceftazidime (2.25 mg in 0.1 ml), and an aqueous specimen was sent for pan-bacterial (16S rRNA), pan-fungal (28S rDNA and ITS primer sets), and viral (HSV1, HSV2, VZV) polymerase chain reaction (PCR) analysis. Two days following intravitreal antibiotic injection, the aching eye pain subsided and vision started to slowly improve. Topical corticosteroids were added 3 days after the intravitreal antibiotic injection. One week after presentation, the aqueous pan-bacterial PCR result returned as positive for *Turicella otitidis*, with all other testing negative. Topical corticosteroids were slowly tapered as ocular inflammation improved. Four weeks after intravitreal antibiotic injection, the patient’s visual acuity had returned to baseline and there was no ocular inflammation on twice daily prednisolone acetate 1% eye drops right eye (Fig. 1b).

## Discussion and conclusion

*Turicella otitidis* is a non-fermenting *Coryneform* gram-positive bacillus almost exclusively isolated from ear exudates and rarely found in skin flora [11]. Its pathogenicity in otitis media has been controversial, but *Coryneform* species are increasingly recognized as important pathogens in granulomatous mastitis [12], and *Turicella otitidis* specifically has been implicated as the causative agent in isolated cases of mastoiditis, cervical abscess, and bacteremia [13]. To our knowledge, this is the first reported case of *Turicella otitidis* endophthalmitis. The patient’s presentation with granulomatous keratic precipitates is consistent with *Turicella otitidis*, since *Coryneform* species have been shown to survive in lipid-filled vacuoles surrounded by a reactive granulomatous infiltrate [12].

*Turicella otitidis* classically shows good susceptibility to ß-lactams, vancomycin and fluoroquinolones [11, 13]. Endophthalmitis due to *Corynebacterium* species in general has been shown to be susceptible to intravitreal vancomycin [6]. In this report, post-injection endophthalmitis secondary to *Turicella otitidis* appeared to be indolent in nature with a rapid response to empiric intravitreal vancomycin and ceftazidime. One interesting practical aspect of this case is the delay in presentation, consistent with the indolent nature of the organism. Although most bacterial endophthalmitis cases associated with intravitreal injections present within 1 week of the injection, the subject in this case did not present until 4 weeks after the injection. It is important to consider a bacterial etiology for post-intravitreal injection endophthalmitis even with a delay in presentation.

Prior work shows that blood culture and PCR have a higher diagnostic yield for endophthalmitis than conventional plate culture in vitreous specimens [14], and blood culture requires a larger volume specimen than PCR. Aqueous specimens typically are smaller in volume than vitreous specimens, but obtaining an aqueous specimen is technically easier than obtaining a vitreous specimen, and a diagnostic anterior chamber paracentesis may be less likely than a vitreous tap to cause an associated retinal detachment. Previous studies show that false positive rates for both aqueous and vitreous PCR are very low [15, 16], and also in this case the granulomatous uveitis and the rapid response to intravitreal antibiotics prior to corticosteroid treatment are confirmatory of the aqueous PCR result.

Pan-bacterial PCR detects whether bacteria-specific DNA encoding 16S ribosomal RNA is present in the specimen; if the 16S ribosomal RNA gene is present, sequences within that gene are compared to DNA sequence databases to identify the specific bacterial organism [17, 18]. In most cases of presumed infectious endophthalmitis after intravitreal injection, routine cultures are used for organism identification. However, for this case with delayed presentation, because the differential diagnosis was broad, the aqueous specimen volume was insufficient for bacterial and fungal cultures as well as viral PCR. PCR of the aqueous fluid proved helpful by identifying *Turicella otitidis* as the causative organism while ruling out more sinister etiologies, such as a fungal endophthalmitis – which was considered given the indolent nature of the endophthalmitis – or a viral retinitis such as acute retinal necrosis unrelated to intravitreal injection. This report demonstrates the utility of PCR in identifying organisms in cases that are more likely to be culture negative, such as when a smaller volume aqueous specimen is obtained.

In summary, we present a case of post-intravitreal injection endophthalmitis that was determined to be bacterial despite the delayed presentation. The causative organism, *Turicella otitidis*, was diagnosed by aqueous pan-bacterial PCR, and the endophthalmitis showed an excellent response to intravitreal antibiotics.

## Data Availability

All data from the case has been included, including images. Any further material is available upon request.

## Abbreviations

AMD: Age-related macular degeneration
CNV: Choroidal neovascularization
PCR: Polymerase chain reaction
VEGF: Vascular endothelial growth factor
